# Supplementary material for: Crystal structures of the mitochondrial deacylase Sirtuin 4 reveal isoform-specific acyl recognition and regulation features
Source: Nat Commun. 2017 Nov 15;8:1513. doi: 10.1038/s41467-017-01701-2 (PMC5686155; doi:10.1038/s41467-017-01701-2)
Supplement: Supplementary file 1 — Supplementary Information [file 41467_2017_1701_MOESM1_ESM.pdf]

Supplementary Figures

a

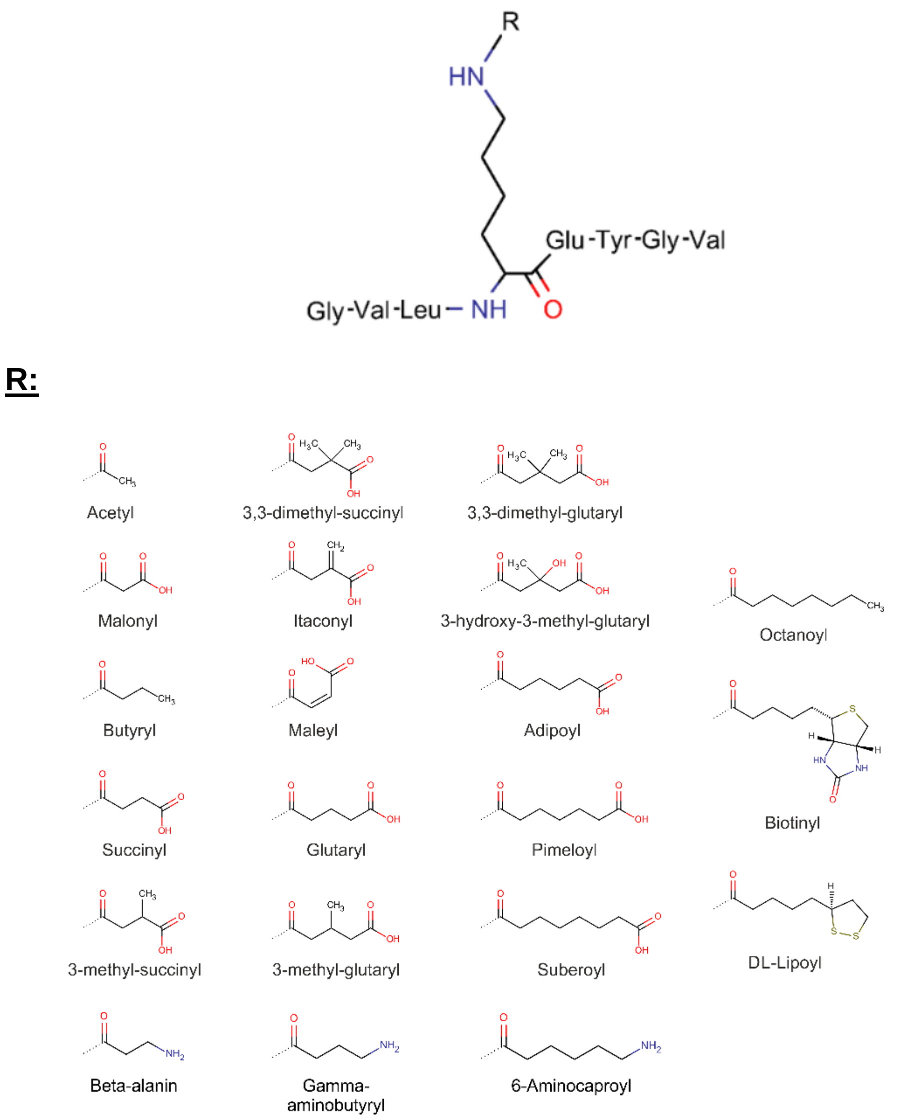

b

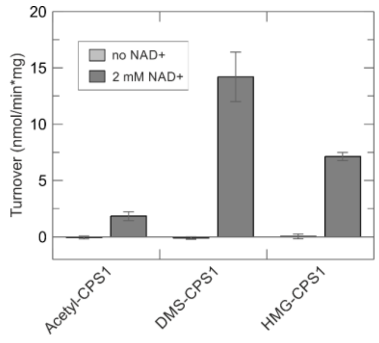

c

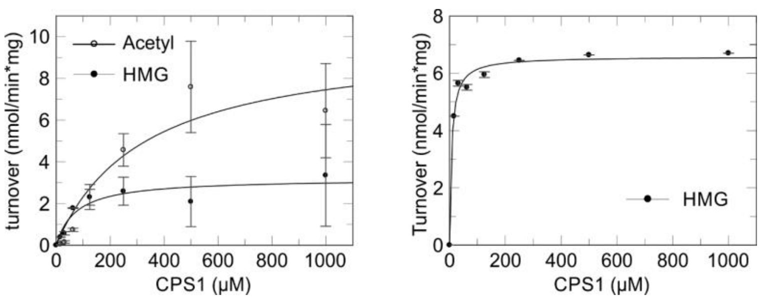

d

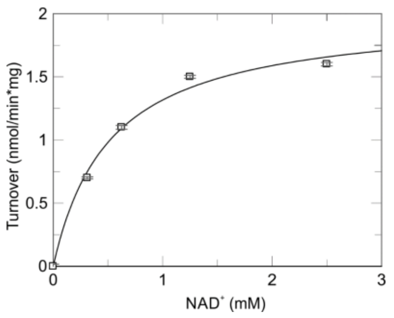

e

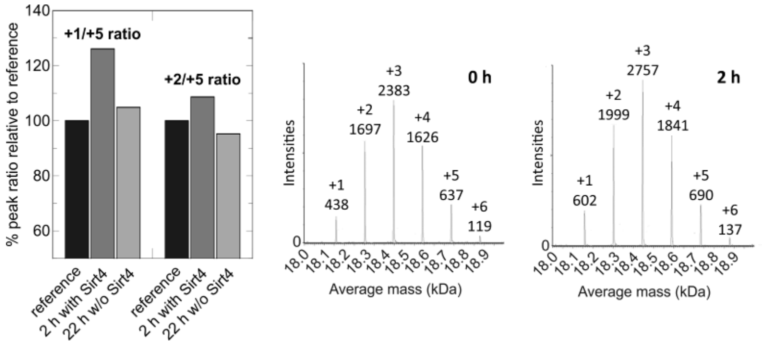

Supplementary Figure 1. Enzymatic activity of Sirt4. **a,** Peptide acyl library for Sirt4 activity screening. A peptide derived from human CPS1 (residues 524-531) was modified with the given acyls (R) on its lysine ε-amino group (covalent linkage indicated by dotted lines). **b,** hSirt4-dependent deacylation reactions with acetyl-, DMS- and HMG-CPS1 peptide substrate performed in presence and absence of 2 mM NAD^+ to confirm their NAD^+ dependency. (n=2; error bars: s.d.) **c,** Analysis of Sirt4-dependent de-HMG-ylation and deacetylation by MS detection of substrate and product peptide (left). The identical de-HMG-ylation analyzed in the coupled enzymatic assay is shown on the right. (n=2; error bars: s.d.) **d,** NAD^+ titration of the hSirt4-dependent de-HMG-ylation of HMG-CypA as a substrate protein. Error bars indicate the correlation coefficient of the linear fit of assay progression. (n=2; error bars: s.d.) **e,** Intact protein MS analysis of Sirt4-dependent de-HMG-ylation of HMG-CypA. The ratios (left) of lower to higher HMG-modification levels (numbered in spectra on the right) increased in presence of Sirt4 but not in control reactions without enzyme.

a

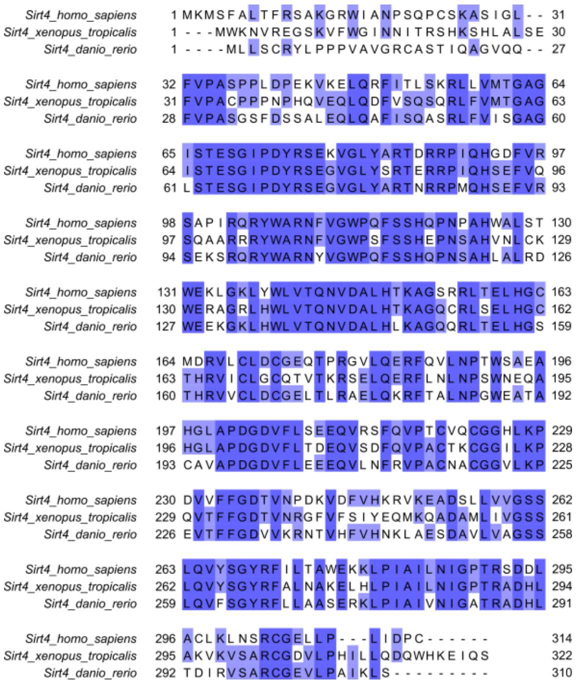
Sirt4_homo_sapiens 1 MKMSFALTFRSAKGRWIANPSQPCSKASIGL-- 31
Sirt4_xenopus_tropicalis 1 ---MWKNVREGSKVFWGINNITRSHKSHLALSE 30
Sirt4_danio_rerio 1 ----MLLSCRYLPPPVAVGRCASTIQAGVQQ-- 27
Sirt4_homo_sapiens 32 FVPASPPLDPEKVKELQRFITLSKRLLVMTGAG 64
Sirt4_xenopus_tropicalis 31 FVPACPPPNPHQVEQLQDFVSQSQRLFVMTGAG 63
Sirt4_danio_rerio 28 FVPASGSFDSSALEQLQAFISQASRLFVISGAG 60
Sirt4_homo_sapiens 65 ISTESGIPDYRSEKVGLYARTDRRPIQHGDFVR 97
Sirt4_xenopus_tropicalis 64 ISTESGIPDYRSEGVGLYSRTERRPIQHSEFVQ 96
Sirt4_danio_rerio 61 LSTESGIPDYRSEGVGLYARTNRRPMQHSEFVR 93
Sirt4_homo_sapiens 98 SAPIRQRYWARNFVGWPQFSSHQPNPAHWALST 130
Sirt4_xenopus_tropicalis 97 SQAARRRYWARNFVGWPSFSSHEPNSAHVNLCK 129
Sirt4_danio_rerio 94 SEKSRQRYWARNYVGWPQFSSHQPNSAHLALRD 126
Sirt4_homo_sapiens 131 WEKLGKLYWLVTQNVDALHTKAGSRRLTELHGC 163
Sirt4_xenopus_tropicalis 130 WERAGRLHWLVTQNVDALHTKAGQCRLSELHGC 162
Sirt4_danio_rerio 127 WEEKGKLHWLVTQNVDALHLKAGQQRLTELHGS 159
Sirt4_homo_sapiens 164 MDRVLCLDCGEQTPRGVLQERFQVLNPTWSAEA 196
Sirt4_xenopus_tropicalis 163 THRVICLGCQTVTKRSELQERFLNLNPSWNEQA 195
Sirt4_danio_rerio 160 THRVVCLDCGELTLRAELQKRFTALNPGWEATA 192
Sirt4_homo_sapiens 197 HGLAPDGDVFLSEEQVRSFQVPTCVQCGGHLKP 229
Sirt4_xenopus_tropicalis 196 HGLAPDGDVFLTDEQVSDFQVPACTKCGGILKP 228
Sirt4_danio_rerio 193 CAVAPDGDVFLEEEQVLNFRVPACNACGGVLKP 225
Sirt4_homo_sapiens 230 DVVFFGDTVNPDKVDFVHKRVKEADSLLVVGSS 262
Sirt4_xenopus_tropicalis 229 QVTFFGDTVNRGFVFSIYEQMKQADAMLIVGSS 261
Sirt4_danio_rerio 226 EVTFFGDVVKRNTVHFVHNKLAESDAVLVAGSS 258
Sirt4_homo_sapiens 263 LQVYSGYRFILTAWEKKLPIAILNIGPTRSDDL 295
Sirt4_xenopus_tropicalis 262 LQVYSGYRFALNAKELHLPIAILNIGPTRADHL 294
Sirt4_danio_rerio 259 LQVFSGYRFLLAASERKLPIAIVNIGATRADHL 291
Sirt4_homo_sapiens 296 ACLKLNSRCGELLP---LIDPC------ 314
Sirt4_xenopus_tropicalis 295 AKVKVSARCGDVLPHILLQDQWHKEIQS 322
Sirt4_danio_rerio 292 TDIRVSARCGEVLPAIKLS--------- 310

b

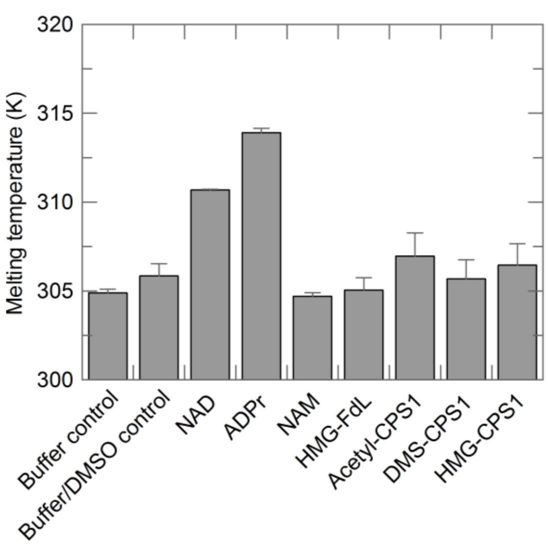
Melting temperature (K)
300
305
310
315
320
Buffer control
Buffer/DMSO control
NAD
ADPr
NAM
HMG-FdL
Acetyl-CPS1
DMS-CPS1
HMG-CPS1

c

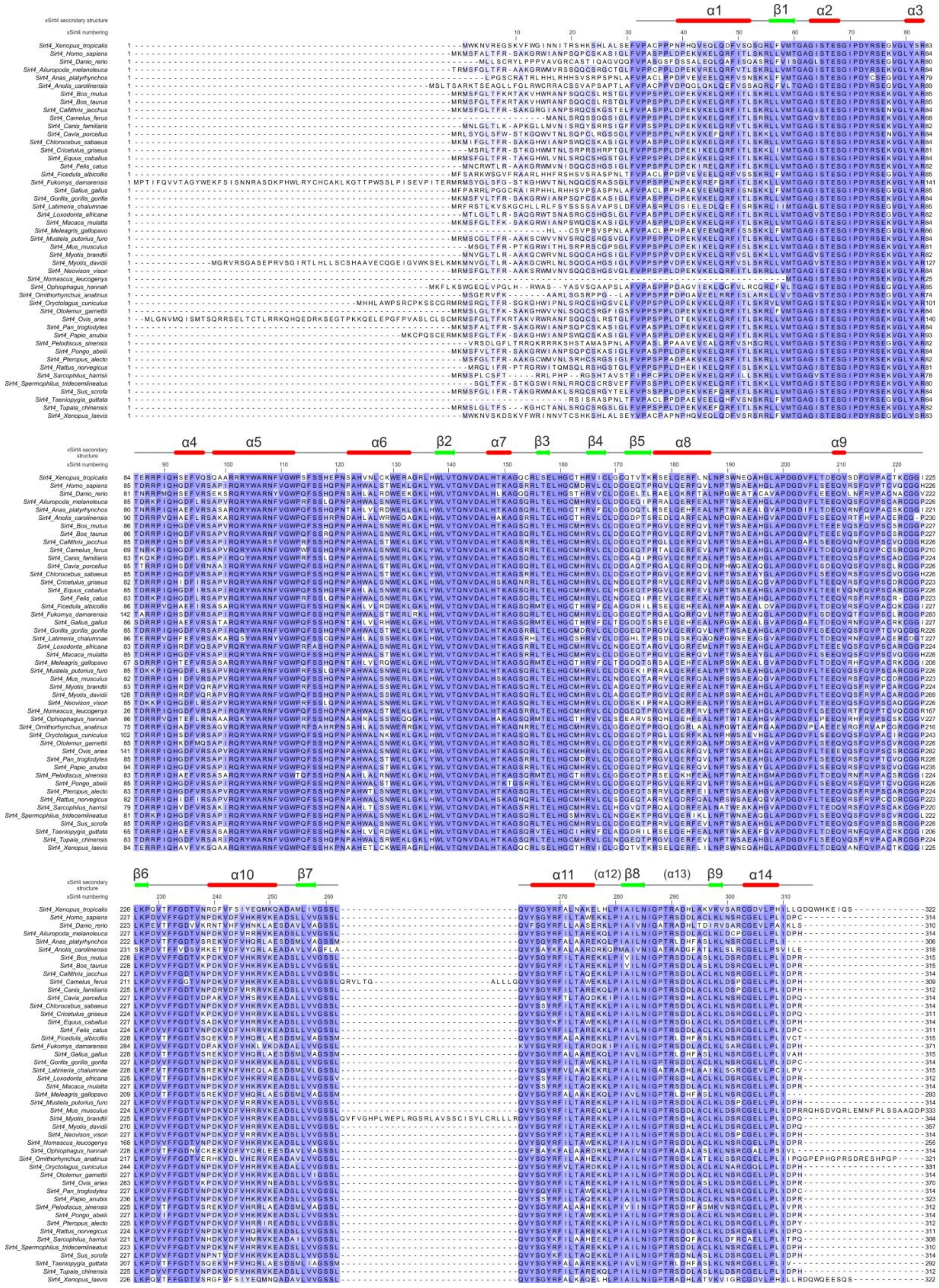

d

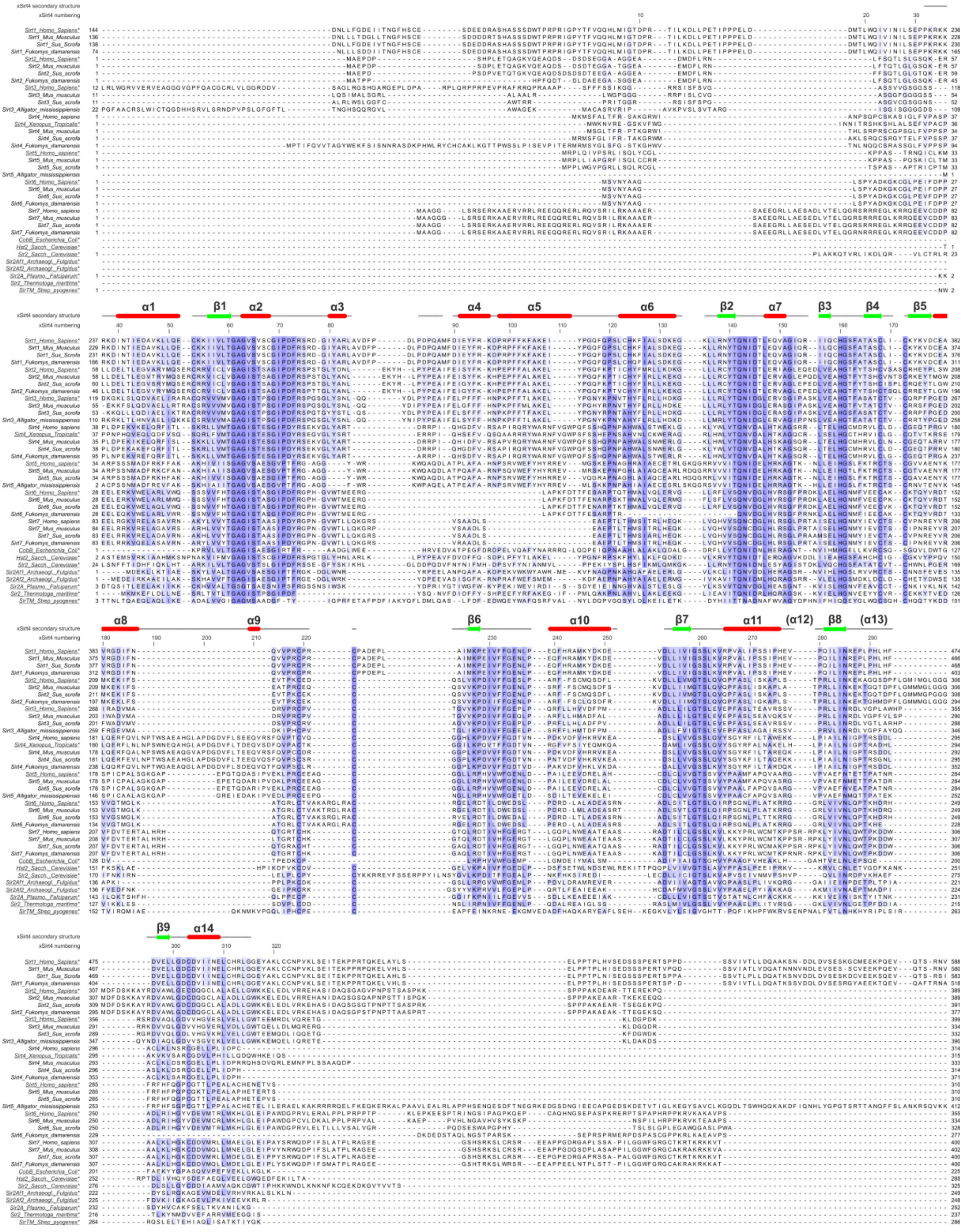

e

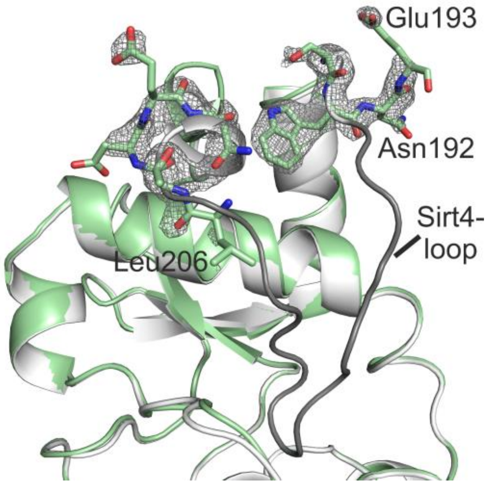

Supplementary Figure 2. Sirt4 conservation and structure. **a,** Sequence alignment of hSirt4, xSirt4 and zSirt4 showing high conservation except for the N-terminal MLS. The coloring encodes conservation (BLOSUM62 score). **b,** Melting temperatures of xSirt4 with various ligands in thermal denaturation shift assays. HMG-FdL, CPS1 peptide, and DMSO control contained 10% DMSO. (n=2; error bars: s.d.) **c,** Multiple sequence alignment of all chordate Sirt4 sequences available from UniProt. The coloring encodes conservation (BLOSUM62 score). Numbering and secondary structure elements are from xSirt4.

d, Structure-based sequence alignment of the catalytic cores of Sirt1-7 and bacterial sirtuins. Our xSirt4/ADPr complex, the most similar structures of human Sirt1,2,3,5,6 and of the bacterial sirtuins (determined using PDBeFOLD) were aligned, the alignment refined manually according to conserved secondary structure elements, and subsequently all chordate sequences of Sirt1-7 available from UniProt were added. Sequences of the structurally characterized sirtuins (highlighted by underlining and *) and four representative sequences of Sirt1-7 are shown. Coloring indicates conservation (BLOSUM62 score). Numbering and secondary structure elements are from xSirt4. **e,** Crystal structure of an xSirt4/thioacetyl-ADP-ribose complex (green). The Sirt4-loop features a different conformation than in the Sirt4/ADPr complex (grey) and is largely undefined as indicated by the 2Fo-Fc electron density contoured at 1σ (gray mesh).

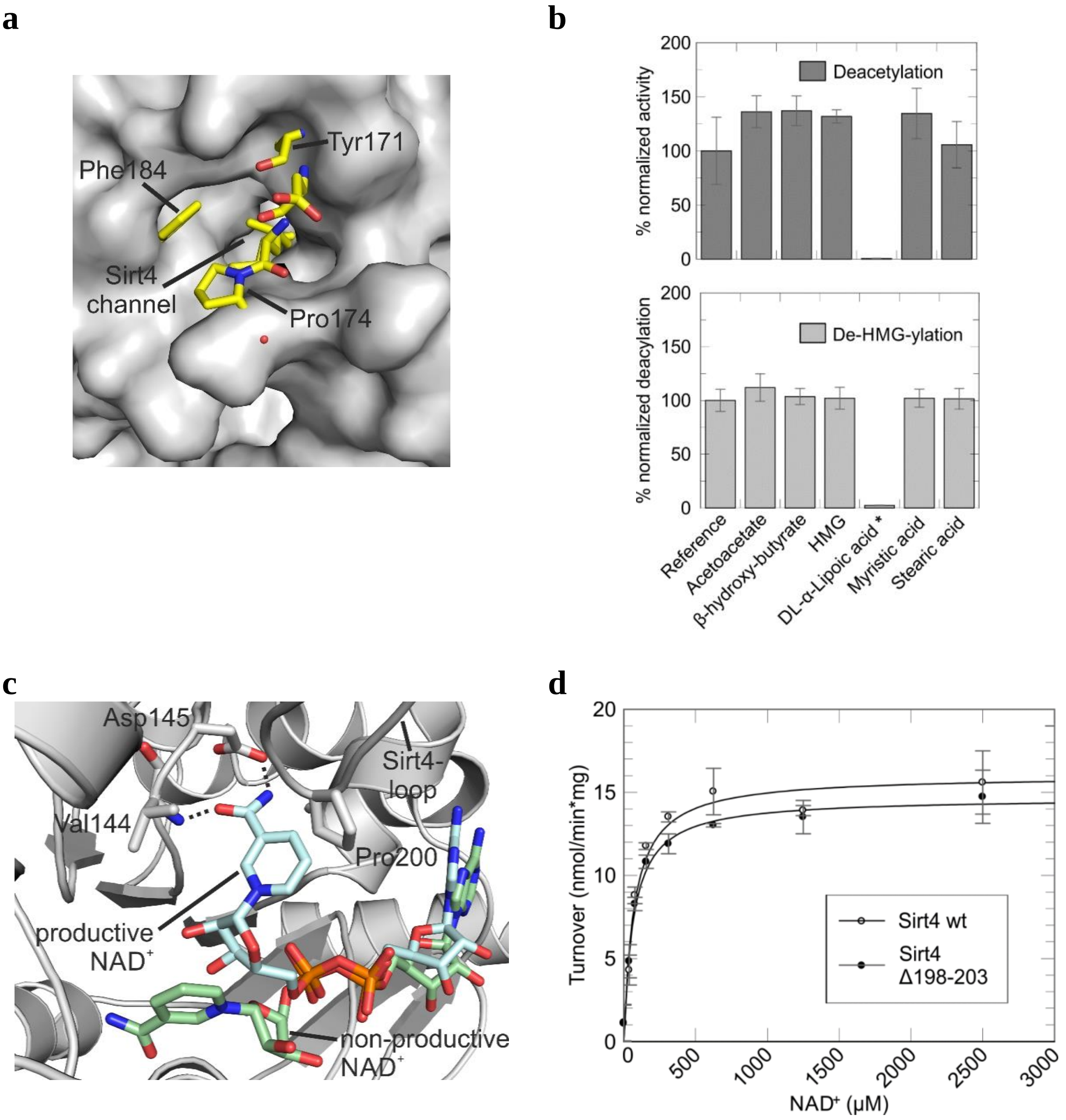
a
Tyr171
Phe184
Sirt4
channel
Pro174
b
Deacetylation
% normalized activity
200
150
100
50
0
De-HMG-ylation
% normalized deacylation
200
150
100
50
0
Reference
Acetoacetate
β-hydroxy-butyrate
HMG
DL-α-Lipoic acid *
Myristic acid
Stearic acid
c
Asp145
Sirt4-
loop
Val144
Pro200
productive
NAD⁺
non-productive
NAD⁺
d
Turnover (nmol/min*mg)
20
15
10
5
0
Sirt4 wt
Sirt4
Δ198-203
0
500
1000
1500
2000
2500
3000
NAD⁺ (μM)

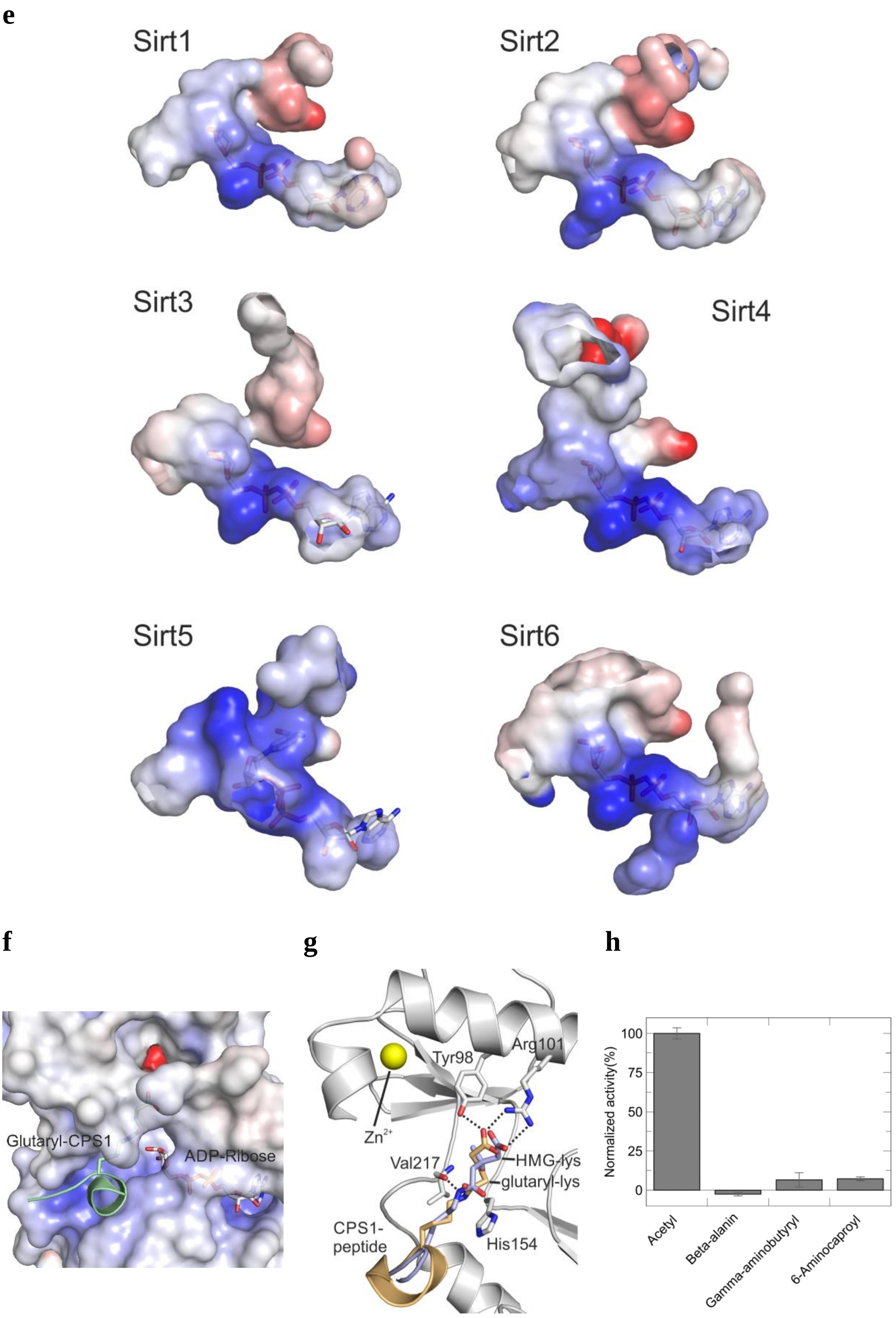
e
Sirt1
Sirt2
Sirt3
Sirt4
Sirt5
Sirt6
f
Glutaryl-CPS1
ADP-Ribose
g
Tyr98
Arg101
Zn2+
Val217
HMG-lys
glutaryl-lys
CPS1-peptide
His154
h
Normalized activity(%)
100
75
50
25
0
Acetyl
Beta-alanin
Gamma-aminobutyryl
6-Aminocaproyl

Supplementary Figure 3. Active site features. **a,** Overlay of xSirt4 (grey surface) and as a representative of other isoforms Sirt3 (PDB ID 3GLR; yellow sticks), showing that the Sirt4 channel is blocked in other isoforms. **b,** Effect of metabolites on xSirt4-dependent deacetylation (top) and de-HMGylation activity (bottom). Metabolites were tested in the coupled enzymatic assay, except for lipoic acid (*****), which was incompatible and therefore examined in MS-based assays. (n=2; error bars: s.d.) **c,** Overlay of xSirt4 (grey cartoon and sticks) with NAD^+ in productive conformation (cyan sticks) from an overlaid Sir2Tm complex (PDB ID 4BUZ) and non-productive NAD^+ (green) from an overlaid Sirt3 complex (PDB ID 4BV3). **d,** NAD^+ titrations for xSirt4 wildtype (wt) and Sirt4-loop deletion mutant (Δ198-203) with HMG-CPS1 peptide substrate. (n=2; error bars: s.d.) **e,** Inner surfaces of the nucleotide binding sites of Sirt1-6, colored by electrostatic potential calculated with APBS2.1 in PyMOL using PDB IDs 4KXQ (Sirt1), 5D7O (Sirt2), 4BN4 (Sirt3), our xSirt4 structure, PDB IDs 3RIY (Sirt5) and 3K35 (Sirt6). ADPr complexes were used for comparability, except for Sirt5 (Sirt5/ADPr complex in open conformation) where we used a Sirt5/succinyl-H3K9/NAD^+ complex. ADPr/NAD^+ are shown as sticks. The surfaces are colored according to electrostatic potential (red/-15 to blue/+15 k_BT/e). **f,** xSirt4 surface colored by electrostatic potential calculated using APBS2.1 in PyMOL (red/-15 to blue/+15 k_BT/e). ADP-ribose is shown as white sticks, and a glutaryl-CPS1 peptide from an overlaid zSirt5 complex (PDB ID 4UTR) is shown in pale green. **g,** Overlay of the active sites of the zSirt5/HMG-CPS1 complex (white, light-blue ligand) with zSirt5/glutaryl-CPS1 (orange; PDB ID 4UTR). Polar contacts are indicated by dashed lines. **h,** xSirt4-dependent deacylation of CPS1-K527 peptides carrying modifications with a positive charge at the distal end (acyl structures shown in **Supplementary Fig. 1a**). Activities are normalized to acetyl-CPS1 substrate. (n=2; error bars: s.d.)

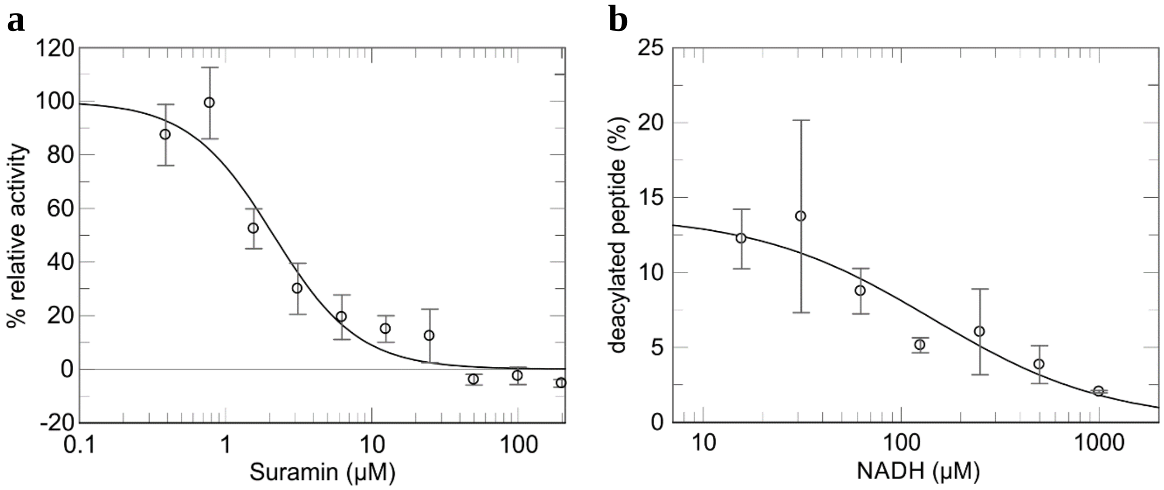

Supplementary Figure 4. Sirt4 inhibition. **a,** Suramin titration to determine the IC_{50} for hSirt4 in the FdL-like de-HMG-ylation assay. (n=2; error bars: s.d.) **b,** NADH-dependent Sirt4 inhibition analyzed with HMG-CPS1 substrate in the MS-based assay. (n=2; error bars: s.d.)

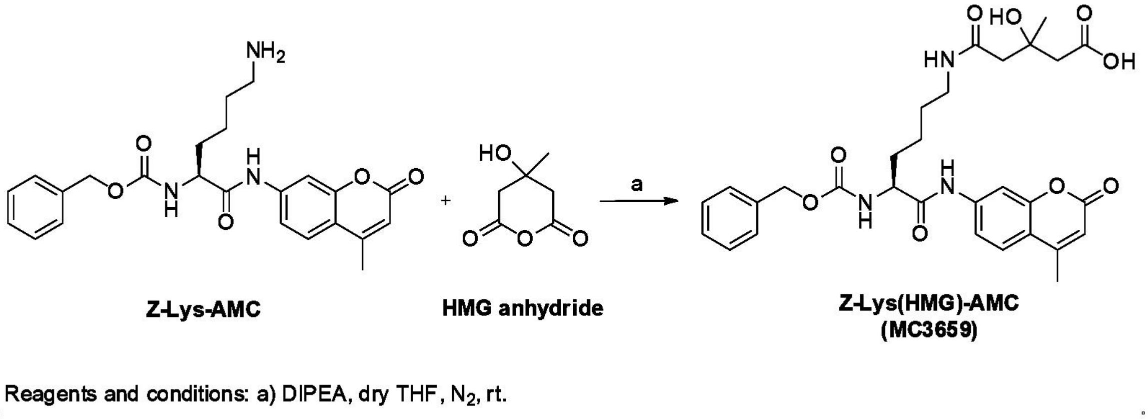

Supplementary Figure 5. Reaction scheme for synthesis of the fluorogenic Sirt4 substrate Z-Lys(HMG)-AMC (HMG-FdL).

Supplementary Table 1 – Primer sequences

Primer	Sequence
hSirt4_25_BamHI_forward	TATAGGATCCTCGAAAGCCTCCATTGGGTTATT
hSirt4_314_HindIII_reverse	TATAAAGCTTTCAGCATGGGTCTATCAAAG
xSirt4_21_NdeI_forward	ATACATATGAGCCACAAATCCCACCTTGCATTGTCAG
xSirt4_32_NdeI_forward	ATACATATGGTCCCTGCATGTCCCCCACCAAATC
xSirt4_315_XhoI_reverse	TATACTCGAGCTATTGGTCCTGTAGCAATATGTGTGGCAATAC
xSirt4_D201A_forward	GTTGGCTCCCGCGGGTGATGTCTTTCTGAC
xSirt4_D201A_reverse	TGTACCCAACCGAGGGCGCCCACTACAGAA
xSirt4_D203A_forward	AGGGCTACCACGCCAGAAAGACTGTCTAC
xSirt4_D203A_reverse	AGGGCTACCACGCCAGAAAGACTGTCTAC
xSirt4_Y73F_forward	GGAATCCCAGACTTTCGCTCAGAAGGG
xSirt4_Y73F_reverse	CCCTTCTGAGCGAAAGTCTGGGATTCC
xSirt4_R101A_forward	GAGTCAGGCTGCAGCACGGAGATATTGGG
xSirt4_R101A_reverse	CCCAATATCTCCGTGCTGCAGCCTGACTC
xSirt4_Y104F_forward	GCTGCAAGACGGAGATTTTGGGCTCGTAACTTTG
xSirt4_Y104F_reverse	CAAAGTTACGAGCCCAAAATCTCCGTCTTGCAGC
xSirt4_R107A_forward	CGGAGATATTGGGCTGCGAACTTTGTAGGATG
xSirt4_R107A_reverse	CATCCTACAAAGTTCGCAGCCCAATATCTCCG
xSirt4_Y104F-R107A_forward	CAGGCTGCAAGACGGAGATTTTGGGCGGCGAACTTTGTAGGATGGCCTAG
xSirt4_Y104F-R107A_reverse	CTAGGCCATCCTACAAAGTTCGCCGCCCAAAATCTCCGTCTTGCAGCCTG
xSirt4_N108A_forward	GAGATATTGGGCTCGTGCGTTTGTAGGATGGCCTAG
xSirt4_N108A_reverse	CTAGGCCATCCTACAAACGCACGAGCCCAATATCTC
xSirt4_Δ189-214_forward	GGTTTCTGAATCTCAATGGATCTAGTCAGGTCCCAGCCTGTACCAAGTG
xSirt4_Δ189-214_reverse	GGCTGGGACCTGACTAGATCCATTGAGATTCAGAAACCTTTCCTGCAGC
xSirt4_Δ192-212_forward	CTCAATCCATCCTGGGGTAGTTCTGACTTTCAGGTCCCAGCCTGTACC
xSirt4_Δ192-212_reverse	GGGACCTGAAAGTCAGAACTACCCCAGGATGGATTGAGATTCAGAAAC
xSirt4_Δ196-205_forward	CCTGGAATGAGCAGGCACTGACAGATGAGCAGG
xSirt4_Δ196-205_reverse	CCTGCTCATCTGTCAGTGCCTGCTCATTCCAGG
xSirt4_Δ196-205+GSS_forward	CAATCCATCCTGGAATGAGCAGGCAGGCTCTTCTCTGACAGATGAGC
xSirt4_Δ196-205+GSS_reverse	GTCTGACACCTGCTCATCTGTCAGAGAAGAGCCTGCCTGCTCATTCC
xSirt4_Δ198-203_forward	GAGCAGGCACATGGGGTCTTTCTGACAGATG
xSirt4_Δ198-203_reverse	CATCTGTCAGAAAGACCCCATGTGCCTGCTC
zSirt4_29_NdeI_forward	ATACATATGGTTCCTGCAAGTGGCTCCTTTGACTCCAG
zSirt4_310_XhoI_reverse	TATACTCGAGTCAGGACAGTTTAATGGCTGGCAGCACTTCTCC
